# A Model for Transgenerational Imprinting Variation in Complex Traits

**DOI:** 10.1371/journal.pone.0011396

**Published:** 2010-07-14

**Authors:** Chenguang Wang, Zhong Wang, Jiangtao Luo, Qin Li, Yao Li, Kwangmi Ahn, Daniel R. Prows, Rongling Wu

**Affiliations:** 1 Department of Statistics, University of Florida, Gainesville, Florida, United States of America; 2 Center for Statistical Genetics, Pennsylvania State University, Hershey, Pennsylvania, United States of America; 3 Department of Statistics, West Virginia University, Morgantown, West Virginia, United States of America; 4 Department of Pediatrics, University of Cincinnati College of Medicine, Cincinnati, Ohio, United States of America; Ohio State University Medical Center, United States of America

## Abstract

Despite the fact that genetic imprinting, i.e., differential expression of the same allele due to its different parental origins, plays a pivotal role in controlling complex traits or diseases, the origin, action and transmission mode of imprinted genes have still remained largely unexplored. We present a new strategy for studying these properties of genetic imprinting with a two-stage reciprocal F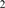 mating design, initiated with two contrasting inbred lines. This strategy maps quantitative trait loci that are imprinted (i.e., iQTLs) based on their segregation and transmission across different generations. By incorporating the allelic configuration of an iQTL genotype into a mixture model framework, this strategy provides a path to trace the parental origin of alleles from previous generations. The imprinting effects of iQTLs and their interactions with other traditionally defined genetic effects, expressed in different generations, are estimated and tested by implementing the EM algorithm. The strategy was used to map iQTLs responsible for survival time with four reciprocal F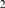 populations and test whether and how the detected iQTLs inherit their imprinting effects into the next generation. The new strategy will provide a tool for quantifying the role of imprinting effects in the creation and maintenance of phenotypic diversity and elucidating a comprehensive picture of the genetic architecture of complex traits and diseases.

## Introduction

Many traits important to agriculture, biology, and human health are complex in terms of the genetic machineries that determine trait formation and development. Broadly speaking, these machineries are equipped with a web of actions and interactions of numerous DNA sequence polymorphisms, modified or altered by environmental factors. To elucidate a detailed picture of the genetic architecture of complex traits, various molecular, statistical, and computational tools have been developed and used in the mapping and identification of specific genes underlying the traits [1]–[8]. The biological basis for developing these tools is that variation in phenotypic traits is due to the changes of DNA sequences in particular regions of the genome and, thus, by analyzing the linkage or association between the genotype and phenotype, significant genes can be detected. More recently, a growing body of new evidence has indicated that chromatin variation, such as differential DNA methylation, independent of DNA sequence changes, may play an important role in regulating the phenotypic formation and progression of complex traits [9]–[12]. Examples of these findings include a spontaneous epigenetic change in the SBP-box promoter leading to the inhibition of fruit ripening in tomatoes [13], the imprinted expression of the axin-fused (Axin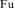) allele resulting in kinked tails in mice [14], and a global loss of cytosine methylation during aging in mice, rats, and humans [15].

To describe variation among individuals in the number or distribution of methylated nucleotides at specific gene sequences, a new term, called epialleles, has been coined [16]. Because epiallele phenotypes can have identical underlying DNA sequences, the genetic control mechanisms of these phenotypes are likely to differ from those estimated from traditional models of quantitative genetics. Thus, it is crucial to screen for epiallelic variants within a population and disentangle epigenetic from more standard genetic sources of phenotypic variance, such as additive genetic variance, dominance variance, epistasis and maternal genetic effects [17]. More recently, Johannes et al. [12] developed a panel of epigenetic Recombinant Inbred Lines (epiRILs) in the reference plant *Arabidopsis thaliana* to identify the genetic variation due to epiallelic variants in flowering time and plant height. Epiallelic variation can also be studied by tracing parent-dependent differences of the same allele. If the same allele functions differently, depending on which parent the allele is derived from, a phenomenon known as genetic imprinting or parent-of-origin effect, this allele may be epigenetic. Previous studies have suggested that genetic imprinting results from an epigenetic mark of differential methylation set during gametogenesis [18]–[20], forming part of the genetic architecture involved in the formation, development, function, and evolution of complex traits and diseases [21]–[25].

The past several years have witnessed an intense interest in mapping and identifying the regions of the genome that contain imprinted sequence variants with genome-wide linkage and association studies. Cheverud et al. [26] and Wolf et al. [27] used a three-generation F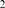 design to map genome-wide imprinted quantitative trait loci (iQTLs) that affect body weight and growth in mice, and they found that these traits may be controlled by QTLs with more complex and diverse effect patterns than previously assumed. Li et al. [28] proposed a reciprocal backcross design to estimate the distribution of iQTLs and quantify their effects on physiological traits related to endosperm development in maize. By modeling alleles identical-by-descent in a multi-generational pedigree of canines, Liu et al. [29] derived a linkage-based random effect to genome-wide scan for the existence of iQTLs that affect canine hip dysplasia. However, there is limited knowledge about whether imprinted effects are inherited over generations and, if yes, how imprinting inheritance takes place [19], [30]–[37]. An understanding of these question will help to characterize the impacts of imprinting loci on the genetic diversity of a biological trait or process [38]–[40].

In this article, we develop a novel strategy for identifying imprinted genes and understanding the transgenerational changes of their effects with a three-generation pedigree. This pedigree is initiated by reciprocally crossing two contrasting inbred lines, leading to two different F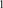 families. The F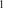 males and females from the same and different families are further crossed to generate four F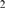 families. Thus, the inheritance of alleles at a gene from a male or female parent can be traced by observing the segregation of the gene in different families. A joint likelihood model is constructed to formulate the effect of imprinted genes on a complex trait. Traditional quantitative genetic theory is integrated to define the effects of imprinting genes (due to the parent-dependent expression of an allele), their interactions with other genetic effect sources (such as additive, dominant, and epistatic), and their generation-dependent actions. We implement the EM algorithm to estimate different genetic effects of imprinted genes and their changes across generations. A testing procedure is proposed to study the pattern of transgenerational imprinting inheritance. The statistical behavior of the model is examined through simulation studies and its usefulness validated from a real data analysis in a three-generation pedigree of mice.

## Methods

### Mating Design

Suppose there are two inbred lines that are sharply contrasting in a complex trait. Each line can serve as a maternal and paternal parent, thus allowing a reciprocal cross. An F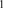 family is produced by mating a dam from one parental line with a sire from the other line, while a reciprocal F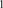 family produced by using the dam and sire from the opposing lines. According to traditional Mendel's first law, these two F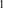 families should be genetically identical. However, if there is an imprinting effect, the two families will be different. Here, we assume that these two F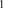 families are epigenetically different. The females and males from the same F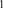 families are crossed to produce two epigenetically “inbred” F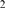 families, whereas those from the opposing F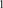 families are crossed to produce two epigenetically “outbred” F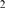 families. Using a quantitative trait locus (QTL) with two alleles 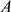 and 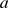, the mating design involving the original parents, reciprocal F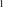 families, and reciprocal F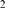 families is illustrated in Figure S1.

Assume that each F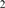 family is typed for the same panel of molecular markers and phenotyped for the same trait of interest. Linkage analysis with these markers allows the construction of an integrative linkage map that covers the genome by combining the four F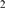 families. The map is then used to identify imprinted quantitative trait loci (iQTLs) that control the trait. The model presented in this article enables geneticists to map iQTLs by combining the segregation pattern of an iQTL in the four different F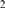 populations.

### Quantitative Genetic Model

Using the iQTL demonstrated in Figure S1, we formulate quantitative genetic models of an iQTL that affects a complex trait. Two inbred lines are reciprocally crossed to generate two F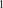 configurations, 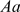 and 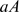, with the same allele inherited from different parents. These two F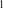 configurations will perform differently if this iQTL shows a significant imprinted effect in the F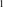 generation. Reciprocal crosses with these F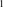 configurations lead to four F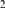 combinations, 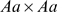, 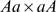, 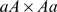, and 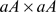, each of which will have the same group of segregating QTL genotypes/configurations, 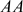, 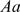, 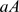, and 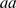. The imprinted effect of the iQTL is inherited into the next generation if two F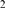 configurations, 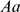 and 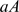, are still different. To test whether this imprinted effect is inheritable and how much it is inherited, we will need to quantify the difference of the imprinted effect of the iQTL expressed in the F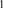 and F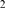 generations. To do that, we attributed the differences among the F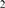 genotypes to two different sources:

1. The same QTL genotype is different from different mating types due to the genetic imprinting of the F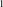 generation. For example, F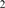 genotype 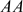 from 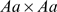 is different than F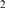 genotype 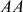 from 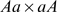 because of the imprinting effect of the F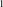 male parent formed in the cross of original inbred lines;
2. F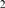 configurations 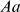 and 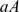 from the same mating type are different because of genetic imprinting formed in the cross of F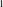 individuals (F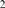).

Thus, a final genotypic value of an F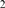 genotype is determined by the imprinting effects of the iQTL in the F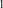 and F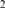 generations, additive and dominance effects, and their interactions. Genotypic values of four F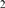 configurations at the iQTL from different mating types are decomposed into different components expressed in Table 1. The component parameters are sorted into seven different groups:

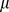
 is the overall mean of all the F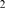 populations,

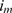
 and 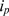 are the imprinting effects of iQTL expressed by the F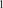 maternal and paternal parents, respectively,

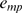
 is the interaction between 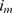 and 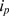,

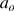
, 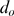, and 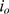 are the additive, dominant, and imprinting effects of the iQTL formed in the F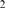,

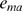
, 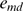, and 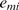 are the interaction effects between the imprinting effects of the F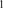 maternal parent and additive, dominant, and imprinting effects expressed in the F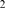, respectively,

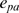
, 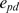, and 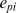 are the interaction effects between the imprinting effects of the F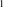 paternal parent and additive, dominant, and imprinting effects expressed in the F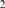, respectively,

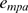
, 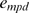, and 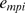 are the interactions between 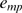 and 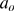, 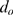, and 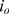, respectively.

**Table 1:** Genetic components of 16 F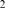 configurations derived from two successive reciprocal crosses.

No.	Mating Type	F 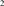 Generation	
		Configuration	Genotypic Value
1	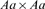	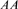	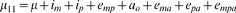
		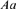	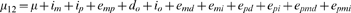
		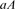	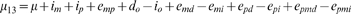
		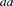	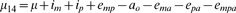
2	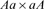	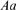	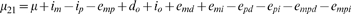
		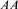	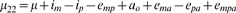
		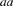	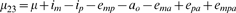
		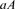	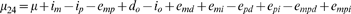
3	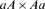	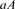	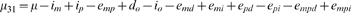
		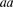	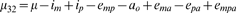
		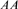	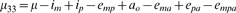
		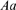	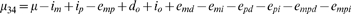
4	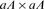	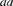	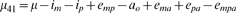
		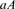	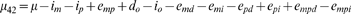
		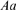	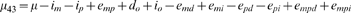
		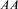	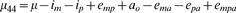

### Mixture Likelihood

The four epigenetically different F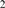 families (Table 1) are observed for a complex trait with respective sample sizes 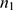, …, 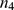. Let 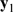, …, 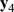 denote the phenotypic values of the trait for different families. An iQTL for the trait that is segregating in four F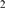 populations can be mapped with interval mapping. Consider a pair of markers between which the iQTL for the trait is hypothesized to be located. The configurations of the iQTL are unobserved, but can be inferred from the genotypes of the markers that bracket the QTL. This inference needs the construction of a likelihood based on a mixture model. Such a likelihood combines the information from four F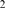 families, expressed as
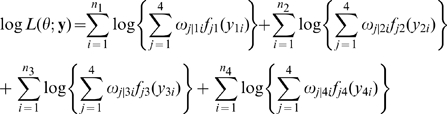
(1)where 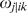 is the conditional probability of an iQTL configuration 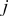 (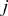 = 1 for 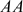, 2 for 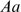, 3 for 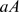, and 4 for 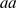) given the marker genotype of individual 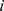 from F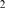 family 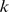 (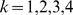), and 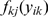 is the normal distribution function of the trait with iQTL configuration-specific mean (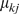) and variance (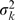). In Wu et al. [41], the procedure for deriving these conditional probabilities are given in terms of the recombination fractions between the left marker and QTL, QTL between the right marker, and the two markers. The EM algorithm was implemented to estimate the genotypic means and variance from the mixture model (1) (see Methods S1).

### Hypothesis Tests

To determine whether there is an iQTL for the complex trait can be tested with log-likelihood ratio approaches. We first tested whether a significant QTL exists in the four F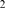 populations using the following null hypothesis,
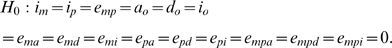
(2)The log-likelihood ratio calculated under the null and alternative hypotheses is compared with the critical threshold determined from permutation tests [42].

After a significant QTL is determined, then the imprinting effect of the QTL can be tested using the following null hypothesis,
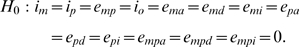
(3)The rejection of null hypothesis (3) implies that the QTL has an accumulative imprinting effect expressed in different generations, which includes main and interaction effects related with genetic imprinting. The imprinting effects expressed in the F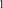 and F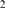 are tested by the null hypotheses, respectively,
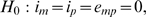
(4)

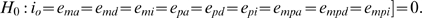
(5)The interactions between the imprinting effect expressed in the F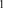 maternal or paternal parents and the additive, dominant, and imprinting genetic effects in the F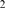 can also be tested, respectively, by
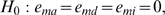
(6)

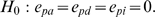
(7)The higher-order interactions among the maternally- and paternally-expressed genetic imprinting in the F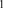 and the additive, dominant, and imprinting genetic effects in the F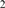 are tested by the null hypothesis,
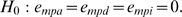
(8)All the genetic effects in equations (3)–(8) can be tested individually. The log-likelihood ratios for hypothesis tests related with genetic imprinting can be thought of being asymptotically 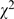-distributed.

## Results

### Worked Example

The newly developed model was used to analyze a data set from a large-scale QTL analysis project in which mice serve as a model system to study survival time to hyperoxic acute lung injury (HALI) [43]. In a screen of 18 inbred mouse strains, C57BL/6J (B) mice were selected as sensitive and 129X1/SvJ (S) mice resistant, based on total survival time in 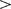
95% oxygen (hyperoxia). Reciprocal F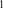 (B
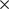
S and S
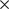
B) mice demonstrated a significant difference in acute lung injury survival time, suggesting possible occurrence of parent-of-origin effects. To further identify specific loci displaying a imprinting effect, both pairs of reciprocal F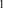 crosses were bred to generate 840 F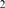 mice, including 213 for (B
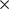
S)
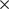
(B
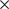
S), 221 for (B
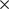
S)
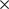
(S
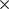
B), 197 for (S
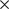
B)
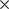
(B
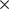
S), and 209 for (S
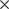
B)
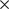
(S
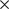
B). A genome-wide linkage map was constructed by typing 93 microsatellite markers located on the 19 autosomes and X-chromosome for four F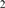 populations of mice derived from sensitive B and resistant S strains.

Phenotype differences between the F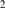 crosses further support possible existence of imprinted genes that affect HALI. By scanning over the linkage map with the log-likelihood ratio test statistics calculated from hypothesis (2), the number and distribution of QTLs for HALI are detected (Figure S2), which is consistent with the discoveries by traditional interval mapping [43]. Five significant QTLs were located between Mit236 and Mit478 on chromosome 1, Mit196 and Mit17 on chromosome 4, Mit116 and Mit145 on chromosome 4, Mit289 and Mit355 on chromosome 9, and Mit175 and Mit5 on chromosome 15. Given their long genetic distance, two significant peaks on chromosome 4 were thought to carry different QTLs. At each of the detected QTLs, the 15 genetic effect parameters including the imprinting, additive, and dominant effects and their interactions across generations, as defined in Table 1, were estimated (Table 2). All these estimated parameters were tested for imprinting effects at different levels. The first test was made for the overall imprinting effects and their interactions expressed in both generations F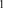 and F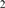, including 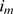, 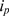, 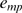, 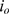, 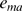, 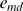, 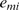, 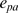, 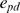, 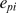, 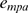, 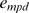, and 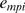. It is found that all the detected QTLs are highly significant for the overall imprinting effects, with the 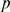-values ranging from 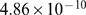 to 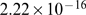 (Table 3). Therefore, these QTLs are regarded as iQTLs.

**Table 2:** Maximum likelihood estimates of genetic effect parameters for each iQTL detected on different chromosomes.

Para-meters	Chromosome				
	1	4	4	9	15
	(Mit236-Mit478)	(Mit196-Mit17)	(Mit116-Mit145)	(Mit289-Mit355)	(Mit175-Mit5)
**Genetic imprinting expressed in the F** 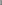					
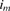	−6.5207	−9.0352	−7.5241	−8.6968	−14.6362
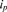	1.6017	0.9479	1.6623	2.7283	11.4645
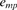	0.6448	0.8244	−0.9077	−1.9431	−7.8337
**Genetic effects expressed in the F** 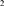					
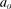	−1.1171	−4.0853	−0.1759	2.1381	11.4756
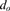	5.5179	−2.7714	1.7809	6.8690	−9.0239
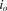	−1.2043	−4.1082	−4.7386	−4.3694	6.0973
**Two-way interactions between genetic effects expressed over generations**					
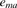	−3.9038	8.7865	3.8376	2.3743	−6.3636
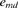	−2.2013	0.5825	−2.2923	0.0604	7.5975
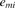	−4.4437	2.4542	2.3924	3.1781	1.3964
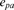	−3.9038	2.6049	8.7361	6.2536	16.7322
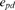	3.5226	4.7608	4.2266	1.1876	−11.7537
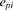	10.6457	−5.4277	−7.1118	−4.2282	−5.0311
**Three-way interactions between genetic effects expressed over generations**					
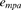	−1.1171	2.0963	−5.0744	−1.7413	−11.6203
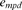	−2.6972	−4.4730	−1.1203	1.4102	8.4446
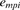	−4.9976	1.1347	0.0192	−0.8235	4.6168

**Table 3:** 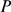
-values for testing the imprinting effects of iQTLs expressed at different levels.

QTL		Test 1	Test 2	Test 3	Test 4	Test 5	Test 6
Chrom.	Marker Interval						
1	Mit236-Mit478	2.22 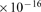	0.0036	3.64 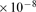	0.2240	0.1406	0.7263
4	Mit196-Mit17	7.93 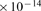	2.26 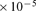	2.63 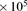	0.6955	0.2073	0.3244
4	Mit116-Mit145	3.30 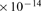	0.0006	4.62 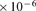	0.4300	0.2143	0.9806
9	Mit289-Mit355	4.86 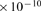	1.60 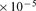	0.0163	0.8872	0.8447	0.9396
15	Mit175-Mit5	1.00 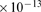	8.90 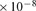	2.21 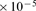	0.1072	0.0213	0.0016

Note: The null hypotheses used are

H_0_: 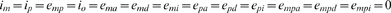 for Test 1.

H_0_: 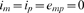 for Test 2.

H_0_: 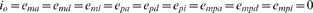 for Test 3.

H_0_: 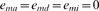 for Test 4.

H_0_: 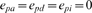 for Test 5.

H_0_: 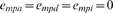 for Test 6.

The second test concerns the imprinting effects expressed in the F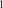 generation by testing whether the paternally- (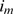) and maternally-imprinted effects (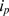) and their interaction (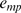) during the cross of the original inbred lines are equal to zero (Table 3). Except for the QTL on chromosome 1 and one QTL on chromosome 4, which are significant at 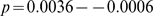, all others display highly significant imprinting effects in the F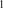 generation (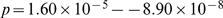). The third test was conducted to see whether there is an imprinting effect in the F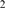 generation by jointly testing the significance of 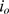, 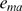, 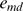, 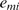, 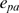, 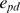, 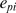, 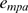, 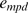, and 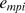. It appears that all the QTLs are highly significant, except for one on chromosome 9 displaying a marginally significant effect. The last three tests focus on the interactions of the imprinting effect in the F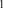 with the additive, dominant and imprinting effects in the F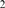. We did not detect many significant interactions between the imprinted effect in the F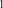 and the overall genetic effects in the F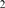, but with two exceptions (Table 3). One is the interaction between the paternally-imprinted effect in the F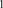 and the overall genetic effects in the F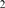 for the QTL on chromosome 15 (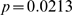), and the other is the three-way interaction among the maternally- and paternally-imprinted effects in the F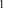 and the overall genetic effects in the F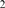 for the same QTL (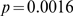).

In sum, all the detected iQTLs show a similar pattern of genetic effect on HALI in the F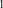 generation, with the maternally-imprinted effect (negative) larger than with the paternally-imprinted effect (positive) (Table 2). Pronounced diversity was observed in the additive and dominant effects among the QTLs when they inherit into the F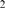 generation. Main imprinting effects in the F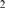 generation were largely reduced, but there is some evidence that imprinted effects are preserved into the F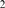 through their interactions with other genetic effects such as additive and dominant.

### Computer Simulation

To examine the statistical behavior of the new model, we performed Monte Carlo simulation studies by mimicking the example of the F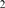 mice. The simulation includes two different parts. In part 1, we simulated 10 evenly-spaced markers in a linkage group of 200 cM. An iQTL is located 35 cM from the first marker at the left. The markers and iQTL are segregating in four reciprocal F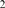 families (Figure S1), initiated with two contrasting inbred lines. The 15 parameters of genetic effects were given and the genotypic values of 16 F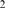 configurations were then calculated. The phenotypic values were then simulated by summing the genotypic values and residual errors assumed to follow a normal distribution with mean zero and variance scaled for different heritabilities 0.10 and 0.40. Two different sample sizes were assumed, i.e., 300 and 500 progeny, for each F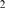 family. All the parameters can be reasonably well estimated with the new model (Table 4). At the modest heritability (0.10), the main imprinting effects and their interactions in the F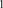 and the main additive, dominant, and imprinting effects in the F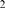 can reasonably well be estimated, even with a smaller sample size (Table 4). To better estimate interactions between imprinting effects of the F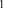 generation and genetic effects of the F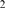, a larger sample size is needed. All parameters can be more precisely estimated when the heritability increases from 0.1 to 0.4. The precise estimation of three-way interactions of imprinting effects between different generations requires a large sample size (2000 in total) and large heritability (0.4).

**Table 4:** Maximum likelihood estimates (and their standard errors) of genetic effect parameters from simulated data under different sample sizes (300 and 500) and heritabilities (0.1 and 0.4).

Parameters	True Value	300		500	
		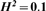	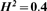	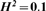	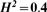
**Genetic imprinting expressed in the F** 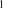					
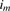	0.15	0.152 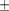 0.0664	0.1519 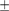 0.0278	0.1466 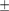 0.0472	0.1501 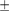 0.0233
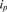	0.15	0.154 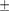 0.0678	0.1534 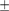 0.0273	0.1450 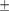 0.0478	0.1482 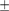 0.0203
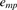	0.1	0.090 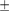 0.0694	0.0990 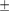 0.0291	0.0954 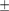 0.0505	0.1005 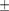 0.0234
**Genetic effects expressed in the F** 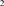					
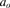	0.3	0.334 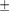 0.1173	0.2947 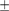 0.0479	0.3199 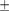 0.0960	0.2943 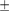 0.0370
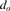	0.6	0.612 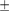 0.0934	0.5982 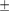 0.0394	0.5940 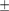 0.0804	0.5992 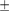 0.0303
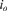	0.2	0.244 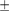 0.1106	0.19660 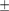 0.0450	0.2300 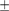 0.0986	0.19820 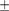 0.0357
**Two-way interactions between genetic effects expressed over generations**					
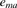	0.04	0.041 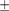 0.1106	0.04081 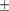 0.0402	0.0425 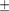 0.0947	0.04201 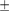 0.0349
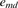	0.04	0.038 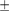 0.1041	0.03758 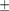 0.0430	0.0441 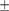 0.0775	0.03828 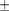 0.0351
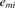	0.04	0.022 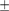 0.1086	0.04262 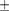 0.0397	0.0153 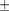 0.0871	0.04022 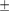 0.0324
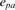	0.04	0.048 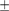 0.1026	0.03688 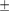 0.0429	0.0415 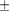 0.0884	0.04118 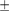 0.0349
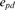	0.04	0.034 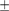 0.0969	0.03574 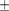 0.0409	0.0463 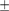 0.0741	0.04274 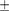 0.0290
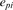	0.04	0.020 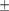 0.1153	0.04290 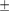 0.0421	0.0193 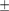 0.0832	0.04130 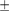 0.0347
**Three-way interactions between genetic effects expressed over generations**					
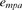	0.04	0.005 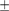 0.1130	0.0461 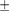 0.0451	0.0295 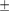 0.0995	0.0467 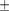 0.0373
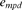	0.04	0.059 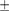 0.0952	0.0406 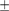 0.0416	0.0470 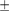 0.0760	0.0385 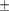 0.0335
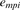	0.04	0.092 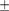 0.1102	0.0353 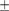 0.0464	0.0753 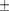 0.0923	0.0342 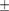 0.0378

In part 2, the simulation was used to test the power of the new model and its false positive rates. The conditions used for power calculation were the same as described above. Table 5 tabulates the results from three different simulation scenarios. There is full power for the detection of overall genetic imprinting effects even when the heritability and sample size are modest (Test 1, Scenario I). Also, great power (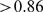) was detected for the overall genetic imprinting effects expressed in the F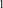 generations (Test 2, Scenarios I and II). Yet, to detect the genetic imprinting expressed in the F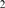, a larger sample size (2000 in total) is needed to achieve a power of 0.99 (Test 3, Scenario II). Much larger heritabilities and/or sample sizes are needed for detecting the interactions between the imprinting effects in the F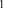 and genetic effects in the F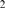, especially when the values of these interactions are small (Tests 4–6, Scenario I). The false positive rates of the estimation for genetic effects by the new model were calculated by simulating the data assuming the absence of those effects (see Scenarios II and III). In general, false positive rates are low for overall genetic imprinting effects (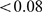) (Test 1, Scenario III), regardless of different heritabilities and sample sizes. Also, false positive rates for overall genetic imprinting effects expressed in the F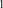 are reasonably low (Test 2, Scenario III). Genetic imprinting effects expressed in the F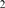 generation, as well as interactions between the imprinting effects of the F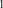 and genetic effects of the F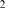, all have very low false positive rates.

**Table 5:** Power and Type I error rates of the model for detecting genetic imprinting effects at different levels.

Scenario	Sample Size	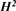	Test 1	Test 2	Test 3	Test 4	Test 5	Test 6
I	300	0.1	100	86	20	3	2	2
		0.4	100	98	33	6	3	4
	500	0.1	100	100	99	22	20	18
		0.4	100	100	100	40	37	32
II	300	0.1	99	98	5	3	2	2
		0.4	100	100	3	4	1	1
	500	0.1	100	100	4	4	1	5
		0.4	100	100	4	2	2	3
III	300	0.1	3	5	2	2	3	1
		0.4	6	6	3	1	3	4
	500	0.1	8	12	7	4	3	4
		0.4	4	7	1	2	2	1

The null hypotheses used are

H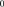: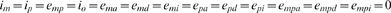 for Test 1.

H_0_: 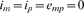 for Test 2.

H_0_: 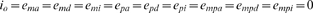 for Test 3.

H_0_: 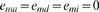 for Test 4.

H_0_: 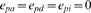 for Test 5.

H_0_: 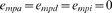 for Test 6.

Three scenarios used are

I. 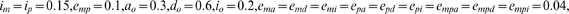

II. 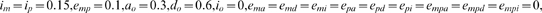

III. 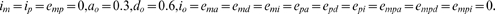.

## Discussion

According to traditional Mendelian genetic theory, the maternally and paternally derived alleles of a gene should have a similar amount of expression because they carry the same DNA sequence. However, a growing number of studies suggest that alleles may be expressed from only one of the two parental chromosomes [18], [44] due to the difference of DNA methylation. Such genetic imprinting or parent-of-origin effects provide a possible source of phenotypic variation for complex traits in the absence of DNA sequence variants [21]–[25]. Thus, to better elucidate the genetic architecture of complex traits and diseases for various organisms including humans, the magnitude and pattern of imprinting effects should be estimated and their impact on quantitative variation quantified.

The attempts to characterize imprinting effects are affected by our incapacity to discern the effect of DNA methylation variants from that of DNA sequence variants using a mapping study. This issue was, however, resolved by comparing two reciprocal crosses in which the maternally- or paternally-derived version of the same allele at a gene can be identified [28], [45]. Liu et al. [29] incorporated identical-by-descent (IBD) sharing into a random-effect mapping model, allowing the characterization of the discrepancy of allelic transmission through different parents. Linkage mapping using controlled crosses or pedigrees with known parents has led to the genome-wide identification of imprinted quantitative trait loci (iQTLs) that affect body weight and growth in mice [26], [27], physiological traits related to endosperm development in maize [28], and hip dysplasia in canines [29].

However, to study the precise genetic mechanisms through which chromatin dynamics alter quantitative variation, a simple test of imprinting effects of iQTLs is not adequate. Rather, a detailed understanding of whether and how imprinting effects are transmitted across generations is crucial for determining the contribution of epigenetic modification to heritable phenotypic variation for a complex trait. In this article, we present a new strategy for estimating and testing imprinting effects of iQTLs and their transgenerational transmission through two-generation reciprocal crosses leading to four epigenetically different F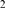 families (Figure S1). The new strategy displays two advantages compared with previous models. First, it provides a comprehensive elucidation of the genetic control mechanisms for a complex trait or disease in terms of traditionally defined additive and dominant effects, newly defined imprinted effects, and their interactions. Second, the strategy has power to detect the changes of imprinting effects from generation to generation, thus facilitating the modeling of transgenerational epigenetic variation and inheritance.

We formulated a mixture model-based likelihood for the imprinting effects of iQTLs flanked by markers in four epigenetically different F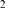 families. A closed form of the EM algorithm was derived to estimate a high-dimensional set of genetic parameters that define the maternally- and paternally-imprinted genetic effects and their interactions in the F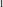, the additive, dominant, and imprinting effects in the F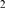, and the interactions of different orders between these effects expressed in different generations. The algorithm was tested through simulation studies from which the minimum heritability and sample size for reasonable estimates of each parameter are determined. Additional simulation studies were performed to test the power for the detection of imprinting effects at different levels. In general, the model shows reasonably low false positive rates for the data in which no imprinting effects exist. In an application of the new model for genetic mapping of iQTL in mice, we identified five significant QTLs on chromosomes 1, 4, 9, and 15 for the overall survival time to hyperoxic acute lung injury (HALI). Each of these QTLs displays remarked imprinting effects on HALI. The model was further used to test when and how these imprinting effects are activated to affect the expression of HALI. In general, all the iQTLs trigger marked imprinting effects in the F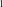 (see 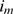 and 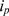 estimates in Table 2). During transmission into the next generation, these imprinting effects were observed to be shrunk (see 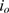 estimates in Table 2). But highly significant imprinting effects in the F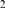 generation can still be detected (Table 3; see also [46]) when the interactions between the imprinting effects of the F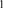 and main effects of the F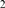 are jointly tested. This result suggests that imprinting effects detected from pure F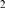 generations, as conducted in [46], may have confounded their interactions with other effects formed during transmission. The results from reanalyzing the mouse data with the new model shed light on the new inheritance and aetiology of HALI.

The model developed in this article will provide a useful tool for studying transgenerational imprinting inheritance and its impact on the variation in complex traits and diseases. As a first attempt of its kind, the model will need to be modified so as to broaden the scope of its application. Given its ubiquitousness in trait control, epistasis between different genes should be incorporated into the current model, helping to draw a comprehensive atlas of the genetic architecture for complex traits. Also, the expression of any genetic effects cannot be isolated from the environment in which organisms are reared [47], [48]. The interactions between different genetic effects and environmental factors should be modeled when a powerful imprinting model is developed. Genetic imprinting may be expressed at the DNA sequence level [49]–[51]. Thus, the integration of haplotype diversity into the model will gain new insights into the genetic control mechanisms of complex traits. All these extensions, although straightforward in theory, will face with an increasing number of parameters being estimated. Statistical explorations for enhancing the efficiency of parameter estimation will be largely demanded. In sum, the development of the new strategy will facilitate our efforts to address many biological questions of fundamental importance in elucidating the genetic architecture of complex traits.

## Supporting Information

Figure S1 A mating design generating four reciprocal F_2_ families, initiated with two inbred lines *AA* and *aa*. The two inbred lines that serve as female (red) and male parents (blue) are crossed reciprocally to generate two F_1_ families. From each of these two families, two progeny, one being a female (red) and the other being a male (blue), are selected to make all possible crosses, leading to four different F_2_ families (with four genotype configurations *AA*, *Aa*, *aA*, and *aa* listed in the box).
(0.04 MB EPS)

Figure S2 The plot of log-likelihood ratio across the mouse genome composed of 19 autosomes and one sex chromosome. Ticks on the x-axis are molecular markers. The peaks of the profile, at which significant QTLs on chromosomes 1, 4, 9, and 15 are detected by the new model, are indicated by arrowed vertical lines. The critical threshold for claiming the existence of significant QTLs is indicated by a horizontal line.
(0.03 MB EPS)

Methods S1 Supporting Methods.
(0.04 MB PDF)
