# Supplementary material for: A Model for Transgenerational Imprinting Variation in Complex Traits
Source: PLoS One. 2010 Jul 14;5(7):e11396. doi: 10.1371/journal.pone.0011396 (PMC2904369; doi:10.1371/journal.pone.0011396)
Supplement: Methods S1 — Supporting Methods. (0.04 MB PDF) [file pone.0011396.s003.pdf]

# Supporting Methods

Here, we describe the EM algorithm implemented to obtain the maximum likelihood estimates (MLEs) of the parameters under the mixture model-based likelihood (1) in the main text. The algorithms are derived separately for the full and reduced model.

## Full Model

The full model means that all 15 effect parameters, as defined in Table 1, are estimated under likelihood (1). In the E step, the posterior probability with which an $F_2$ individual $i$ from mating type $k$ carries iQTL configuration $j$ is calculated as

$$\Omega_{j|ik} = \frac{\omega_{j|ik}(y_{ik})}{\sum_{j'=1}^{4} \omega_{j'|ik} f_{j'k}(y_{ik})}. \tag{S1}$$

In the M step, the genotypic values of an iQTL configuration and variance are estimated by the posterior probability calculated, which is expressed as

$$\mu_{kj} = \frac{\sum_{i=1}^{n_k} \Omega_{j|ki} y_{ki}}{\sum_{i=1}^{n_k} \Omega_{j|ki}} \tag{S2}$$

$$\sigma_k^2 = \frac{\sum_{k=1}^{4} \sum_{i=1}^{n_k} \sum_{j=1}^{4} \Omega_{j|ki}(y_{ki} - \mu_{j|ki})^2}{\sum_{k=1}^{4} n_k} \tag{S3}$$

The E (Equation S1) and M steps (Equations S2 and S3) are iterated until the estimates of parameters converge to stable values. The stable estimates are the MLEs of the parameters. The location of an iQTL is estimated by assuming the iQTL at a series of fixed positions within the marker interval. The maximum value of the likelihood corresponds to the MLE of the iQTL position.

After the genotypic values of iQTL configurations ($\mu_{kj}$) are estimated, genetic component parameters

are estimated using the following equations:

$$
\begin{aligned}
\hat{\mu} &= \tfrac{1}{8}(\hat{\mu}_{11}+\hat{\mu}_{41}+\hat{\mu}_{22}+\hat{\mu}_{32}+\hat{\mu}_{23}+\hat{\mu}_{33}+\hat{\mu}_{14}+\hat{\mu}_{44}) \\
\hat{i}_m &= \tfrac{1}{8}(\hat{\mu}_{11}+\hat{\mu}_{41}+\hat{\mu}_{22}+\hat{\mu}_{32}-\hat{\mu}_{23}-\hat{\mu}_{33}-\hat{\mu}_{14}-\hat{\mu}_{44}) \\
\hat{i}_p &= \tfrac{1}{8}(\hat{\mu}_{11}+\hat{\mu}_{41}-\hat{\mu}_{22}-\hat{\mu}_{32}+\hat{\mu}_{23}+\hat{\mu}_{33}-\hat{\mu}_{14}-\hat{\mu}_{44}) \\
\hat{e}_{mp} &= \tfrac{1}{8}(\hat{\mu}_{11}+\hat{\mu}_{41}-\hat{\mu}_{22}-\hat{\mu}_{32}-\hat{\mu}_{23}-\hat{\mu}_{33}+\hat{\mu}_{14}+\hat{\mu}_{44}) \\
\hat{a}_o &= \tfrac{1}{8}(\hat{\mu}_{11}-\hat{\mu}_{41}+\hat{\mu}_{22}-\hat{\mu}_{32}-\hat{\mu}_{23}+\hat{\mu}_{33}-\hat{\mu}_{14}+\hat{\mu}_{44}) \\
\hat{d}_o &= \tfrac{1}{8}(-\hat{\mu}_{11}+\hat{\mu}_{21}+\hat{\mu}_{31}-\hat{\mu}_{41}+\hat{\mu}_{12}-\hat{\mu}_{22}-\hat{\mu}_{32}+\hat{\mu}_{42}+\hat{\mu}_{13}-\hat{\mu}_{23}-\hat{\mu}_{33}+\hat{\mu}_{43} \\
&\quad -\hat{\mu}_{14}+\hat{\mu}_{24}+\hat{\mu}_{34}-\hat{\mu}_{44}) \\
\hat{i}_o &= \tfrac{1}{8}(\hat{\mu}_{21}-\hat{\mu}_{31}+\hat{\mu}_{12}-\hat{\mu}_{42}-\hat{\mu}_{13}+\hat{\mu}_{43}-\hat{\mu}_{24}+\hat{\mu}_{34}) \\
\hat{e}_{pa} &= \tfrac{1}{8}(\hat{\mu}_{11}-\hat{\mu}_{41}-\hat{\mu}_{22}+\hat{\mu}_{32}-\hat{\mu}_{23}+\hat{\mu}_{33}+\hat{\mu}_{14}-\hat{\mu}_{44}) \\
\hat{e}_{pd} &= \tfrac{1}{8}(-\hat{\mu}_{11}+\hat{\mu}_{21}+\hat{\mu}_{31}-\hat{\mu}_{41}-\hat{\mu}_{12}+\hat{\mu}_{22}+\hat{\mu}_{32}-\hat{\mu}_{42}+\hat{\mu}_{13}-\hat{\mu}_{23}-\hat{\mu}_{33}+\hat{\mu}_{43} \\
&\quad +\hat{\mu}_{14}-\hat{\mu}_{24}-\hat{\mu}_{34}+\hat{\mu}_{44}) \\
\hat{e}_{pi} &= \tfrac{1}{8}(\hat{\mu}_{21}-\hat{\mu}_{31}-\hat{\mu}_{12}+\hat{\mu}_{42}-\hat{\mu}_{13}+\hat{\mu}_{43}+\hat{\mu}_{24}-\hat{\mu}_{34}) \\
\hat{e}_{ma} &= \tfrac{1}{8}(\hat{\mu}_{11}-\hat{\mu}_{41}+\hat{\mu}_{22}-\hat{\mu}_{32}+\hat{\mu}_{23}-\hat{\mu}_{33}+\hat{\mu}_{14}-\hat{\mu}_{44}) \\
\hat{e}_{md} &= \tfrac{1}{8}(-\hat{\mu}_{11}+\hat{\mu}_{21}+\hat{\mu}_{31}-\hat{\mu}_{41}+\hat{\mu}_{12}-\hat{\mu}_{22}-\hat{\mu}_{32}+\hat{\mu}_{42}-\hat{\mu}_{13}+\hat{\mu}_{23}+\hat{\mu}_{33}-\hat{\mu}_{43} \\
&\quad +\hat{\mu}_{14}-\hat{\mu}_{24}-\hat{\mu}_{34}+\hat{\mu}_{44}) \\
\hat{e}_{mi} &= \tfrac{1}{8}(\hat{\mu}_{21}-\hat{\mu}_{31}+\hat{\mu}_{12}-\hat{\mu}_{42}+\hat{\mu}_{13}-\hat{\mu}_{43}+\hat{\mu}_{24}-\hat{\mu}_{34}) \\
\hat{e}_{mpa} &= \tfrac{1}{8}(\hat{\mu}_{11}-\hat{\mu}_{41}-\hat{\mu}_{22}+\hat{\mu}_{32}+\hat{\mu}_{23}-\hat{\mu}_{33}-\hat{\mu}_{14}+\hat{\mu}_{44}) \\
\hat{e}_{mpd} &= \tfrac{1}{8}(-\hat{\mu}_{11}+\hat{\mu}_{21}+\hat{\mu}_{31}-\hat{\mu}_{41}-\hat{\mu}_{12}+\hat{\mu}_{22}+\hat{\mu}_{32}-\hat{\mu}_{42}-\hat{\mu}_{13}+\hat{\mu}_{23}+\hat{\mu}_{33}-\hat{\mu}_{43} \\
&\quad -\hat{\mu}_{14}+\hat{\mu}_{24}+\hat{\mu}_{34}-\hat{\mu}_{44}) \\
\hat{e}_{mpi} &= \tfrac{1}{8}(\hat{\mu}_{21}-\hat{\mu}_{31}-\hat{\mu}_{12}+\hat{\mu}_{42}+\hat{\mu}_{13}-\hat{\mu}_{43}-\hat{\mu}_{24}+\hat{\mu}_{34})
\end{aligned}
$$

Based on the consistency property of the maximum likelihood method, the estimates of unknown parameters derived from the MLEs of other parameters are the MLEs of these unknown parameters.

## Reduced Model

The reduced model is defined as one in which only some of the 15 effect parameters are estimated, letting the remaining equal to zero. In the extreme case in which all the 15 effects are zero, only with the population mean being estimated, no EM algorithm is needed. When one or more than one, but less than 15, effect parameters are estimated, a general procedure is to use the same EM algorithm developed for the full model, but with the constraint(s) that poses on the relationships among genotypic values ($\mu_{kj}$) derived under the condition of the other parameters equal to zero. Here, we incorporate the Augment-M algorithm in which the M step is derived under the reduced model using the following steps:

(1) For individual $i$ from mating type $k$ with observed phenotypic value $y_{ki}$, we augment the trait value $y_{j|ki}$ of that individual that carries iQTL configuration $j$ ($j = 1, ..., 4$), i.e.,

$$y_{j|ki} = \Omega_{j|ki} y_{ki},$$

where $\Omega_{j|ki}$ is the posterior probability obtained from the E-step;

(2) Define a dummy variable $X_{j|ki}$ that meets

$$E(y_{j|ki}) = X_{j|ki}\beta = \frac{1}{\sum_k^4 \sum_i^{n_k} \Omega_{j|ki}} X_j\beta,$$

where $\beta = (\mu, i_m, i_p, e_{mp}, a_o, d_o, i_o, e_{ma}, e_{md}, e_{mi}, e_{pa}, e_{pd}, e_{pi}, e_{mpa}, e_{mpd}, e_{mpi})$ is the genetic effect vector;

(3) Arrange the augmented data in a linear model framework. Then, we have

$$\mathbf{y}_A = \mathbf{X}_A\beta,$$

where $\mathbf{y}_A = \{y_{j|ki}\}$ and $\mathbf{X}_A = \{X_{j|ki}\}$. For a given reduced model, represented by $K'\beta = 0$, we have

$$\hat{\beta}_K = \hat{\beta} - (\mathbf{X}_A'\mathbf{X}_A)^{-1}K[K'(\mathbf{X}_A'\mathbf{X}_A)^{-1}K]K'\hat{\beta}, \tag{S4}$$

where $\hat{\beta} = (\mathbf{X}_A'\mathbf{X}_A)^{-1}\mathbf{X}_A'\mathbf{Y}_A$;

(4) The variance is estimated by

$$\hat{\sigma^2}_K = \frac{1}{N}\sum_k^4 \sum_i^{n_k} \Omega_{j|ki}(Y_{ki} - X_j\hat{\beta}_K)^2, \tag{S5}$$

where $N$ is the total number of individuals from the four $F_2$ families.

The iteration is made between the E step (equation S1) and M step (equations S4 and S5) until the stable values are obtained. These stable values are the MLEs of the parameters under the reduced model.
